# Common Features of Opportunistic Premise Plumbing Pathogens

**DOI:** 10.3390/ijerph120504533

**Published:** 2015-04-24

**Authors:** Joseph O. Falkinham

**Affiliations:** Department of Biological Sciences, Virginia Tech., 1405 Perry Street, Blacksburg, VA 24061, USA; E-Mail: jofiii@vt.edu; Tel.: +1-540-231-5931; Fax: +1-540-231-9307

**Keywords:** opportunistic premise plumbing pathogens, Legionella, Mycobacteria, *Pseudomonas*, biofilm formation, disinfectant-resistance

## Abstract

Recently it has been estimated that the annual cost of diseases caused by the waterborne pathogens *Legionella pneumonia*, *Mycobacterium avium*, and *Pseudomonas aeruginosa* is $500 million. For the period 2001–2012, the estimated cost of hospital admissions for nontuberculous mycobacterial pulmonary disease, the majority caused by *M. avium*, was almost $1 billion. These three waterborne opportunistic pathogens are normal inhabitants of drinking water—not contaminants—that share a number of key characteristics that predispose them to survival, persistence, and growth in drinking water distribution systems and premise plumbing. Herein, I list and describe these shared characteristics that include: disinfectant-resistance, biofilm-formation, growth in amoebae, growth at low organic carbon concentrations (oligotrophic), and growth under conditions of stagnation. This review is intended to increase awareness of OPPPs, identify emerging OPPPs, and challenge the drinking water industry to develop novel approaches toward their control.

## 1. Opportunistic Premise Plumbing Pathogens (OPPPs)

### 1.1. Introduction to OPPPs

Opportunistic premise plumbing pathogens (OPPPs) are microbial residents of drinking water distribution systems and premise plumbing. Premise plumbing includes water pipes in houses, hospitals, condominiums, apartments, and office buildings. OPPPs are pathogens and normal inhabitants of drinking water; they are not contaminants of drinking water but are adapted to growth and persistence in drinking water systems. Unlike contaminants such as *Escherichia coli* and *Salmonella* spp., OPPPs grow in drinking water distribution systems. In fact, OPPP numbers and frequency of isolation do not correlate with indices of fecal contamination of drinking water [1,2]. OPPPs share a number of characteristics that directly contribute to their survival, growth, and persistence in drinking water plumbing systems including: disinfectant-resistance, biofilm-formation, growth in amoebae, growth at low organic carbon concentrations (oligotrophic), and growth under conditions of stagnation.

It is the objective of this brief review to identify the common characteristics of OPPPs, by describing the ecology of a number of exemplary bacteria; including *Legionella pneumophila*, *Mycobacterium avium*, *Pseudomonas aeruginosa*, *Methylobacterium* spp., *Acinetobacter baumanii*, and *Aeromonas hydrophila.* The OPPPs reviewed are human pathogens and depending on the microorganism can result in life-threatening lung (*i.e.*, Legionnaires’ disease) or blood-stream infections (e.g., *Pseudomonas aeruginosa*) or long-term chronic pulmonary disease (e.g., *M. avium*). A recent review documented the enormous cost of OPPP infection—more than $500 million/year [3,4]. The review will identify the key, shared characteristics of well-established OPPPs (e.g., *L. pneumophila*, *M. avium*, and *P. aeruginosa*) to serve as a guide to identify emerging OPPPs (e.g., *Methylobacterium spp. Acinetobacter baumanii*, *Aeromonas hydrophila*, and *Segniliparus* spp.) and to suggest experimental approaches to identify emerging OPPPs and to devise measures that can be employed to control OPPP numbers.

### 1.2. Legionella pneumophila

*L. pneumophila* is likely the most widely known waterborne opportunistic premise plumbing pathogen (OPPP). It causes a life-threatening pneumonia, Legionnaires’ disease, whose source has been traced to water and aerosols. The estimated incidence of Legionnaires’ disease is increasing at a rate of 5% per year and there were approximately 18,000 cases in 2011 [5]. Transmission of *L. pneumophila* occurs via aerosols and can come from humidifiers, showers, or cooling towers. *L. pneumophila* has been isolated or detected (using PCR-based approaches) in samples of drinking water collected across the United States [2]. Both community- and hospital-acquired pneumonias have been described [6]. Although *L. pneumophila* is fastidious and difficult to cultivate, high numbers are found in drinking water samples [2]. *L. pneumophila’s* fastidious requirements for growth seem inconsistent with its presence in drinking water, but can be explained by either its long term survival [7] or its ability to grow in phagocytic amoebae; falling in the category of amoebae-resisting microorganisms (ARM) [8]. In fact, persistence of *L. pneumophila* in drinking water may be due to its growth in amoebae; quite possibly, its presence in drinking water may require its carriage in amoebae. That hypothesis has yet to be directly tested, but if proven that will have a major impact on efforts to control this waterborne pathogen. Specifically, control of *L. pneumophila* could be exerted through reduction in amoebae numbers. Consistent with its presence in drinking water, *L. pneumophila* is relatively chlorine-resistant (Table 1) [9]. Further, water-adapted cells of *L. pneumophila* are 7-fold more resistant to chlorine than are cells grown in laboratory medium [9]. *L. pneumophila* is found in biofilms on a variety of pipe surfaces [10,11]. Coupled with its presence in amoebae and biofilms, the habitats occupied by *L. pneumophila* provide it with protection from disinfection, nutrients for growth, and resistance to being washed out by water flow.

**Table 1:** Chlorine resistance of waterborne pathogens relative to *Escherichia coli*.

Genus or Species	CT_99.9%_^a^	Reference
*Escherichia coli*	0.09 (reference)	Taylor *et al.* (2000) [12]
*Legionella pneumophila*		
Medium-grown	7.5 (83-fold)	Kuchta *et al.* (1985) [9]
Water-adapted	52.5 (580-fold)	Kuchta *et al.* (1985) [9]
*Mycobacterium avium*		
Medium-grown	51 (567-fold)	Taylor *et al.* (2000) [12]
Water-adapted		Steed and Falkinham (2006) [13]
*Pseudomonas aeruginosa*	1.92 (21-fold)	Grobe *et al.* (2001) [14]
*Methylobacterium* spp.	1.5 (16.7-fold)	Furuhata *et al.* (1989) [15]
*Acinetobacter baumanii*	59 (658-fold)	Karumathil *et al.* (2014) [16]
*Aeromonas hydrophila*	2.6 (29-fold)	Sisti *et al.* (1998) [17]

**^a^** Product of concentration (mg/L) and duration of exposure (min) to kill 99.9% of cells.

### 1.3. Mycobacterium avium

*M. avium* is a waterborne opportunistic pathogen that causes pulmonary infection, primarily, but not exclusively, in slender, taller, and older women and men, cervical lymphadenitis in children, and bacteremia in immunodeficient individuals [18]. Pulmonary infections have been tracked by DNA fingerprinting to showerheads [19] and household plumbing [20]. Numbers of *M. avium* increase as drinking water moves from the treatment plant to home [1]. The majority of *Mycobacterium* spp. infections in the United States are caused by *M. avium* and that species serves as a representative of the group of environmental mycobacteria; called the nontuberculous mycobacteria (NTM). Although NTM disease is not required to be reported, estimates based on hospital admissions show that the prevalence of infection is increasing at a rate of between 8% and 10% annually with approximately 30,000 cases in the United States at present [21,22]. *M. avium* grows very slowly (1 generation per day in rich laboratory media), but readily adheres and forms biofilms on pipe surfaces [23], so it is not washed out. *M. avium* is very disinfectant-resistant [12] and that resistance is increased by its residence in biofilms [13]. *M. avium* is relatively resistant to elevated temperature 90% survive exposure to 50 °C (125 °F) for 60 min [24], which likely contributes to its residence in households [20] through their ability to survive and grow in hot water heaters. *M. avium* is also able to tolerate periods of stagnancy, common in premise plumbing, and can grow at 6% and 12% oxygen as well as air (21% oxygen) [25]. Another factor likely contributing to its residence in drinking water systems and premise plumbing is its resistance to killing by phagocytic amoebae [26]. Like other waterborne premise plumbing pathogens, *M. avium* is an ARM [27]. Rather being phagocytozed and killed by amoebae (*Acanthamoeba* spp., *Hartmanella* spp., and *Vermamoeba* spp.), ARM multiply within amoebae [27]. The spectrum of characteristics of *M. avium* make it ideally adapted to drinking water distribution systems and premise plumbing: disinfectant-resistance, growth in drinking water, thermal tolerance, biofilm formation, growth under conditions of stagnation, and resistance to killing by amoebae.

### 1.4. Pseudomonas aeruginosa

*P. aeruginosa* has long been associated with water borne infections, particularly amongst burn patients. Cystic fibrosis patients are at risk for *P. aeruginosa* pneumonia; a difficult to treat infection because of the bacterium’s relative resistance to antibiotics. *P. aeruginosa* is a cause of hospital-acquired as well as community-associated life-threatening pneumonias and it is estimated that approximately 1000 cases per year in the United States [28]. *P. aeruginosa* is relatively resistant to disinfectants used in water treatment such as chlorine (Table 1) [14]. Infections have been linked to *P. aeruginosa* in solutions used for surface sterilization, bronchoscopes washed with non-sterile water or disinfectant solutions, and in-dwelling catheters [29]. Although most commentators discuss the transfer of drinking water pathogens from water to patient, there is evidence of patient introduction of *P. aeruginosa* into water taps [30,31]. Alternatively, it is possible that those cases were due to *P. aeruginosa* in sink traps and the cells were aerosolized by splashing during hand-washing. *P. aeruginosa* readily forms biofilms [32,33], so it can persist and grow in flowing systems (e.g., pipes). Cells of *P. aeruginosa* in biofilms, like other biofilm inhabitants, survive higher dosages of disinfectants, in part due to the presence of layers of cells and extracellular matrix material in the biofilm [32,33]. *P. aeruginosa* can survive periods of stagnation in a water system (e.g., plumbing) due to its ability to utilize nitrate (NO_3_^−^) as a terminal electron acceptor in the absence of oxygen [34]. In drinking water systems disinfected with chloramine, stagnation may result in *P. aeruginosa* proliferation due, in part, to nitrate-driven respiration.

### 1.5. Methylobacterium spp.

*Methylobacterium* spp. are Gram-negative, slowly growing opportunistic pathogens in premise plumbing. Early references to these bacteria that form distinctive pink-pigmented colonies identify these as *Protomonas* or *Pseudomonas*. *Methylobacterium* spp. have been associated with nosocomial infections in individuals with reduced immunity [35,36] and in persons with indwelling catheters [37,38]. Pseudo-outbreaks of *Methylobacterium mesophilica* were associated with use of bronchoscopes that were contaminated by tap water in the room used for preparation of bronchoscopes [39,40]. Methylobacteria are common inhabitants of drinking water having been isolated from water collected in hospitals and households [39,40]. One reason for their residence in drinking water is their chlorine-resistance [15,41,42], a characteristic they share with other premise plumbing pathogens (Table 1). In addition to chlorine-resistance, surface-adherence, biofilm-formation, and desiccation-tolerance are likely contributors to *Methylobacterium* spp. persistence in drinking water systems [43]. It is probable that methylobacteria belong to the group of amoebae-resistant microorganisms (ARM), as *Methylobacterium* spp. have been recovered from amoebae isolated from drinking water systems [27].

### 1.6. Acinetobacter baumanii

Recently, attention has been directed toward *Acinetobacter* spp., in particular *Acinetobacter baumanii* as a result of a large number of *Acinetobacter* infections amongst injured U.S. troops in the Middle East [44]. In addition, *Acinetobacter* spp. have been shown responsible for community-acquired severe, bacteremic pneumonia in which 80% of patients required hospitalization in an intensive care unit [45]. *Acinetobacter* spp. have been reported as present in drinking water samples collected from wells [46] or from treated distribution systems and households [47] and hospitals [48]. As would be expected from an inhabitant of drinking water systems and premise plumbing, *A. baumanii* is resistant to chlorine (Table 1) [16]. Like other opportunistic waterborne premise plumbing pathogens, *A. baumanii* readily adheres to surfaces and forms biofilms [16]. *A. baumanii* is an ARM, with cell numbers increased approximately 10^5^-fold in amoebae-*A. baumanii* co-cultures compared to number increases of amoebae-free *A. baumanii* cultures [49]. Although co-cultures do not accurately capture the growth of intracellular bacteria due to the fact that phagocytosis continues through the co-cultivation, the dramatic increased number of *A. baumanii* cells [49] certainly indicates that *A. baumanii* is an ARM and its numbers are increased in the presence of phagocytic amoebae. In addition to the disinfectant resistance of *A. baumanii*, isolates of that species from either drinking water [47] or hospital water samples [48] are resistant to a wide variety of antibiotics. Thus, this opportunistic premise plumbing pathogen presents challenges in treatment of infections.

### 1.7. Aeromonas hydrophila

*Aeromonas hydrophila* is another member of the drinking water microbiome that causes bacteremia, meningitis, and wound infections [50]. In common with other OPPPs, young children, the elderly, and individuals with lowered immune status are more susceptible [51]. *Aeromonas hydrophila* has been isolated from natural and municipal drinking water samples [51,52,53]. Interestingly, the numbers of *A. hydrophila*-associated gastroenteritis cases have correlated with the number of *A. hydrophila* isolates in a municipal drinking water system [52]. However, numbers of *A. hydrophila* did not correlate with numbers of *Escherichia coli* or fecal coliforms [52]. A substantial proportion (75%) of *A. hydrophila* isolates from drinking water samples express the hemagglutination, hemolytic, and cytotoxic activities that have been shown to be associated with human infection [50,53]. That observation proves that the drinking water isolates are not nonpathogenic variants of an otherwise pathogenic species; always a question concerning an opportunistic pathogen. *A. hydrophila* isolates are found in raw water, even brackish water like *M. avium*, and drinking water distribution systems, particularly in biofilms [54,55,56,57]. Drinking water distribution system biofilms samples were shown to yield yellow-pigmented *A. hydrophila* [57] and *A. hydrophila* was shown to rapidly adhere to a variety of surfaces [54]. *A. hydrophila* is 29-fold more resistant to chlorine than *E. coli* (Table 1) [17] and chlorination of drinking water results in the disappearance of *E. coli*, but not *A. hydrophila*, [52]. Interestingly like *M. avium* [1], *A. hydrophila* numbers in drinking water distribution systems increase between the treatment plant and the ends, a phenomenon called “re-growth” in the drinking water industry [57]. Re-growth clearly shows that a microorganism is able to grow in drinking water despite the presence of a disinfectant residual. 

### 1.8. Segniliparus spp.

*Segniliparus* spp. are a relatively recently described group of bacteria that share many characteristics in common with the environmental mycobacteria; namely they are Gram-negative, stain acid-fast, have very long chain (C_60_–C_100_) mycolic acids, a high GC content of 68%–72%, and grow slowly [58]. *Segniliparus rugosus* has been isolated from patients with cystic fibrosis suffering from deterioration of lung function [59,60], suggesting that *S. rugosus* may be an emerging pathogen of cystic fibrosis patients. A second species, *Segniliparus rotundus* was isolated from a non-cystic fibrosis, non-HIV-infected patient with pneumonia [61] showing that infections of this genus is not limited to cystic fibrosis patients. *S. rugosus* has even been isolated from a California Sea Lion suffering from bronchiolitis [62]. Although there have been no reports of isolation of *Segniliparus* spp. from natural or drinking water samples, the close relatedness to the *Mycobacterium* coupled with its isolation from a California sea lion (*Zalophus californianus*), strongly suggest it will be found to share those characteristics shared by opportunistic premise plumbing pathogens; for example, disinfectant-resistance, biofilm-formation, and resistance to amoebae killing.

### 1.9. Additional Possible Opportunistic Premise Plumbing Pathogens

Although there is insufficient information available currently, *Cronobacter* spp. [63,64], *Stentrophomonas maltophila* [65,66,67], and even *Helicobacter* [68,69,70] are likely OPPPs. *Cronobacter* spp. are Gram-negative bacteria whose infections have been linked to contaminated powdered infant formula, foods, and environmental sources [64], forms biofilms, survives for long periods of time at low temperatures, and is resistant to disinfectants [64]. *S. maltophila* has been recovered from tap water [65] and nosocomial infections have been linked to their presence in hospital water sources [66]. Further, members of the *Stentrophomonas* genus have wide metabolic versatility, are resistant to disinfectants and heavy metals [66] and have been shown to grow in the amoebae *Vermamoeba* (nee *Hartmanella*) *vermiformis* [67]. *Helicobacter pylori* has been isolated from biofilms in drinking water systems [68], forms biofilms [69] and undergoes a morphological transformation to a coccoid form that permits survival in the environment [70].

## 2. Distinguishing Between Premise Plumbing Pathogens and Contaminants in Drinking Water

### 2.1. Common Features of Premise Plumbing Pathogens

First, one feature shared by OPPPs is that they are all opportunistic pathogens; infected individuals have one or more risk factors making them more susceptible than other people to these bacteria. Second, a number of ecologic and physiologic characteristics are shared amongst the waterborne premise plumbing pathogens (Table 2). For example, all are relatively resistant to chlorine (Table 1) and other disinfectants used in water treatment. The consequence is that OPPPs survive exposure to residual disinfectant levels while competitors for nutrients are killed. Thus, it is important to consider a drinking water treatment system as selective, reducing population diversity and allowing the amplification of numbers of a smaller group of microorganisms; here the focus is on opportunistic pathogens. 

Other shared characteristics include: slow growth, biofilm-formation, resistance to killing by phagocytic amoebae, thermal tolerance, and survival under low oxygen levels (stagnation). Slow growth means that these opportunistic pathogens are often missed; for example, it takes up to 7 days at 30 °C for the appearance of *Methylobacterium* spp. colonies and up to 14 days for the first appearance of *M. avium* colonies incubated at 37 °C. Slow growth is actually an advantage because slow growth is associated with slow death. For example, chlorine-resistance of *L. pneumophila* and *M. avium* is increased if cells are adapted to drinking water (Table 1). All OPPPs are found in biofilms and belong to the ARM group of waterborne microorganisms [23]. However, not all of these opportunistic pathogens are equally resistant to disinfectants (Table 1), temperature, and low oxygen. *M. avium* is clearly the most resistant to chlorine and only *P. aeruginosa* can grow under anaerobic conditions. However, the levels for all are high enough for them to survive conditions in drinking water distribution systems and premise plumbing. Another factor shared by OPPPs that distinguishes them from other waterborne microorganisms is their behavior in water. Numbers of contaminants of drinking water such as *Escherichia coli* will fall as they move from the source. That decrease in number is due to dilution by the water and to the absence of growth. However, OPPPs are normal inhabitants of drinking water and their numbers increase after the water leaves the treatment plant [1,2,57]. Further, numbers of OPPPs are not correlated with numbers of *E. coli*, fecal coliforms. fecal streptococci or other measures of microbial water quality.

**Table 2:** Common characteristics of waterborne residential, commercial, and household pathogens.

Common Characteristics of OPPPs
Infection Linked to Drinking Water Exposure
Persistence in Drinking Water
Re-growth in Drinking Water Distribution Systems
Disinfectant (Chlorine)-Resistance
Biofilm-Formation
Thermal-Tolerance
Resistance to Phagocytosis by Amoebae
Survival and Growth at Low Oxygen
Slow Growth

Generally, OPPPs are relatively resistant to antibiotics; though not by the same mechanisms. For example, the thick, wax- and lipid-rich outer membrane of *M. avium* confer resistance to most commonly used antibiotics [71] and *P. aeruginosa* isolates have barriers to the entry of antibiotics [72]. In that these opportunist pathogens grow in amoebae, they are capable of growth in macrophages [73]. Intracellular growth and persistence in macrophages also provides a barrier to entry of antibiotics. Antibiotic-resistance of this group of waterborne pathogens creates problems in developing strategies for treating infections. 

### 2.2. Distinguishing between Premise Plumbing Pathogens and Contaminants 

Several lines of evidence can be used to determine whether an OPPP is present and possibly linked to infection. These include: (1) identification of an OPPP species and evidence of (2) regrowth in a drinking water distribution system of premise plumbing; (3) growth and survival in amoebae (ARM); (4) disinfection-resistance; and (5) biofilm-formation. 

## 3. Control of OPPPs

Although this review focuses on the shared characteristics of OPPPs that does not necessarily mean that a single measure will reduce numbers of OPPPs in drinking water distribution systems and premise plumbing. OPPPs are members of widely different taxonomic groups of bacteria and it would be naïve to consider them as reacting to single measures identically. Clearly, a multi-pronged approach will be required to control OPPPs. Additionally, control measures may not have the same outcomes in treatment plants, distribution systems, and premise plumbing; conditions vary widely. Finally, water utilities are not responsible for water quality issues that develop in premise plumbing. Thus, information must be conveyed to two different communities; utilities and homeowners and building operators. With those constraints in mind, the following list of control measures is offered.

(1) Increased disinfectant concentration is problematic and likely ineffective.
(2) One approach that can be instituted by a water utility is turbidity reduction. As OPPPs prefer surface attachment and biofilm-formation, they are attached to soil particles in natural rivers and lakes. Turbidity reduction reduces *M. avium* numbers [1], and might also reduce numbers of other OPPPs.
(3) For homeowners and building managers, raising hot water heater temperatures may reduce OPPP numbers. High hot water heater temperatures are associated with reduced numbers of *L. pneumophila* [74] and *M. avium* [14].
(4) Disinfectant substitution may lead to unexpected problems. For example, substitution of chlorine by monochloramine to reduce the production of chlorinated organics and to increase efficacy of biofilm-killing, has been shown to result in the disappearance of *L. pneumophila*, but increased recovery of *M. avium* [75].
(5) As it appears that most, if not all OPPPs are amoebae-resisting microorganisms (ARMs), reduction of amoebae numbers may result in a concomitant reduction of OPPPs.

## 4. Conclusions

The emergence of opportunistic premise plumbing pathogens (OPPPs), such as *L. pneumophila*, *M. avium*, and *P. aeruginosa* is one consequence of improvements in water treatment; in particular widespread disinfection and organic carbon reduction. Those improvements have created a selective environment for OPPPs. This review identifies the shared characteristics of OPPPs that result in their persistence and growth in drinking water systems and household plumbing; including disinfectant-resistance, growth at low carbon concentrations, surface-adherence and biofilm-formation, stagnation-resistance, and growth in phagocytic amoebae. Identification of the common characteristics of OPPPs leads, in turn, to the identification of emerging OPPPs, such as *Acinetobacter baumanii* and *Aeromonas hydrophila*. As OPPPs behave differently from classic water-borne pathogens (e.g., *Salmonella*), novel strategies must be developed to reduce their numbers in drinking water.
